# On newly and recently recorded species of the genus *Lema* Fabricius (Coleoptera, Chrysomelidae, Criocerinae) from Taiwan

**DOI:** 10.3897/zookeys.262.4152

**Published:** 2013-02-01

**Authors:** Chi-Feng Lee, Yoko Matsumura

**Affiliations:** 1 Applied Zoology Division, Taiwan Agricultural Research Institute, 189 Chung-Cheng Road, Taichung 41362, Wufeng, Taiwan; 2 Laboratory of Systematic Entomology, Department of Ecology and Systematics, Graduate School of Agriculture, Hokkaido University, Sapporo, Japan; 3 Entomology Group, Institut für Spezielle Zoologie und Evolutionsbiologie mit Phyletischem Museum, FSU Jena, Erbertstrasse 1, 07743 Jena, Germany

**Keywords:** *Lema diversipes*, *Lema lacertosa*, flagellum, genitalia, Insecta, Taiwan

## Abstract

New records of four species (*Lema lacertosa* Lacordaire, 1845, *Lema diversipes* Pic, 1921, *Lema cyanella* (Linnaeus, 1758), *Lema trivittata trivittata* Say, 1824 and additional information on one recently recorded species (*Lema solani* Fabricius, 1798) are reported for Taiwan. *Lema diversipes* Pic, 1921 is removed from synonymy with *Lema lacertosa* Lacordaire, 1845; both species are redescribed. A lectotype is designated for *Lema phungi* Pic, 1924. The synonymies of *Lema phungi* Pic, 1924 and *Lema jeanvoinei* Pic, 1932 with *Lema lacertosa* Lacordaire, 1845 are supported. A revised key to the known species in Taiwan is provided.

## Introduction

*Lema* Fabricius is the largest genus of the subfamily Criocerinae and is distributed worldwide (Monrós 1959, Schmitt 1988). Most members are relatively small in size and good flyers, so it is usually not easy to collect multiple individuals of the same species simultaneously (Vencl et al. 2004). A lot of species were described in the early era of chrysomelid taxonomy, and the descriptions were very brief and mainly based on color patterns (Warchałowski 2011). These factors cause difficulties in the identification of *Lema* specimens to species, which is an encumbrance to phylogenetic and evolutionary work on the group in spite of their interesting morphology (stridulatory organ: Schmitt and Traue 1990, reproductive systems: Matsumura and Suzuki 2008) and ecology (plant-insects interactions: Schmitt 1988, Morton and Vencl 1998, Vencl and Morton 1999a, b, Vencl et al. 2004). Recently, to resolve this situation, the introductory but comprehensive taxonomic works which include Asian or Palaearctic *Lema* species were published (Schmitt 2010, Warchałowski 2010, 2011). Accumulation of faunal information based on reliable identification is desired to establish a robust hypothesis of an evolutionary scenario for *Lema* as the next step.

The Taiwanese islands, the focus of this study, are located in eastern-south Asia and are a subtropical to tropical region. Although taxonomic and/or faunal studies of Taiwan were done by Chûjô (1951), Kimoto and Chu (1996), Kimoto and Takizawa (1997), and Lee and Cheng (2007, 2010), some species were newly found through the effort of the Taiwan Chrysomelid Research Team since 2007. In addition, taxonomic confusion occurs with *Lema lacertosa* Lacordaire, 1845. To resolve it, we studied all available type specimens of the synonyms of *Lema lacertosa*.

## Materials and methods

Most of Taiwanese populations were collected by the Taiwan Chrysomelid Research Team. These specimens are kept in Matsumura’s private collection (Jena, Germany) temporarily, and these will be deposited in the laboratory of Systematic Entomology of Hokkaido University (Japan) at a future date. In addition to these specimens, we used specimens which were borrowed from Muséum National d’Histoire Naturelle (MNHN, Paris, France), the laboratory of Systematic Entomology of Hokkaido University (SEHU, Sapporo, Japan), and the Taiwan Agricultural Research Institute (TARI, Taichung, Taiwan). In the results section, the following symbols are used to describe information on data labels exactly: a slash (/) indicates that words were written on one line of a data label, and double slashes (//) indicates that they were written on another label.

Scanning electronic microscopy (LSM-6510, JEOL) images were captured to observe fine structures in detail. To observe male and female genitalia, firstly we softened dried specimens by warming them in distilled water over night (50–60°C). Then, the abdomen was removed from the body, and softened in KOH solution (ca. 5-10%) for two days. Then we observed the genitalia under the microscope (Olympus SZX12) and drew them.

**Terminology.** The terminology for the exoskeleton is based mainly on Chûjô (1951), the male genitalia on Sharp and Muir (1912), and the female reproductive organs on Suzuki (1988). For descriptions of internal-sac sclerites we followed the terminology established by Tishechkin et al. (2011). Because Tishechkin et al. (2011) named sclerites based on the position during copulation (internal sac inverted), their “dorsal” and “ventral” sclerites correspond to “ventral” and “dorsal” sclerites of Matsumura and Yoshizawa (2012) who named each sclerites based on the position in repose.

## Results

### Lema (Lema) diversipes

Pic, 1921 stat. res.

http://species-id.net/wiki/Lema_diversipes

Lema diversipes Pic, 1921: 3 [Yunnan] (Paris); Monrós 1959: 185; treated in Gressitt and Kimoto 1961: 69 (synonym of *Lema lacertosa*).

#### Type series.

Holotype ♀: “Pe Yen Tsing/ Yunnan// Lema // voir Mouhoti Baly // diversipes/ n sp// type // MUSEUM PARIS / 1958/ coll. M. Pic // HOLOTYPE (red label) // MNHN/ EC2232”.

#### Material examined.

**Taiwan**: 22 exs.: Taipei: Shihting, 11.VIII.2007, leg. M.-H. Tsao (6 exs. in SEHU; 16 exs. in TARI).

#### Diagnosis.

*Lema diversipes* can be separated from *Lema lacertosa* by the following combination of characters: body is distinctly larger and relatively stout in shape; anterior margin of the clypeus is waved and protruded slightly; posterior lines of the vertex grove slightly curved; almost all of the ventral surface is black; sternites covered by pubescence, except the posterior margins of the sterna 1-4 glabrous.

#### Redescription.

**Figures 1–4.**
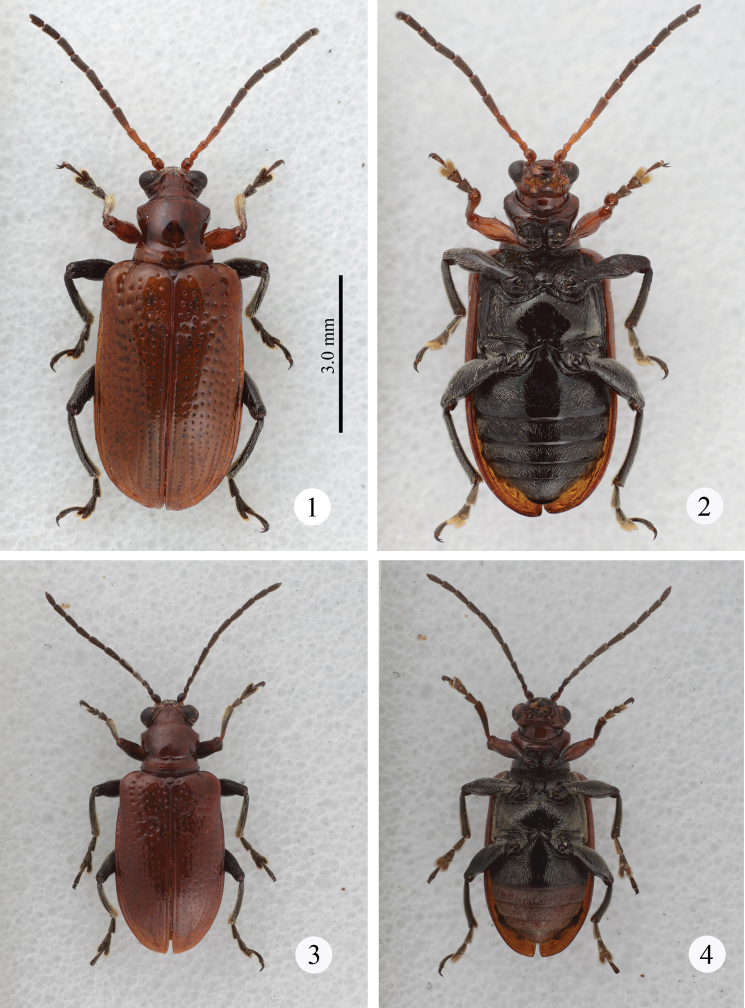
Habitus. **1–2** *Lema diversipes* **1** dorsal view **2** ventral view **3–4** *Lema lacertosa* **3** dorsal view **4** ventral view. (all at the same scale)

**Body coloration** (Figs 1–2). Dorsum (holotype): Head reddish-brown except for black apex of mandible, antenna yellowish-brown; pronotum and elytra reddish-brown, scutellum brown; prolegs orange except for coxae which are brownish-orange, mesolegs blackish-brown, metalegs black. Dorsum (Taiwanese individuals): Antennomeres 1–4 orange, 5–11 blackish-brown; profemur and tibia orange, procoxae, trochanter, tarsi and claws blackish-brown. Venter: prosternum orange; meso- and metasterna black; abdomen blackish-brown. Pubescence white.

**Figures 5–14.**
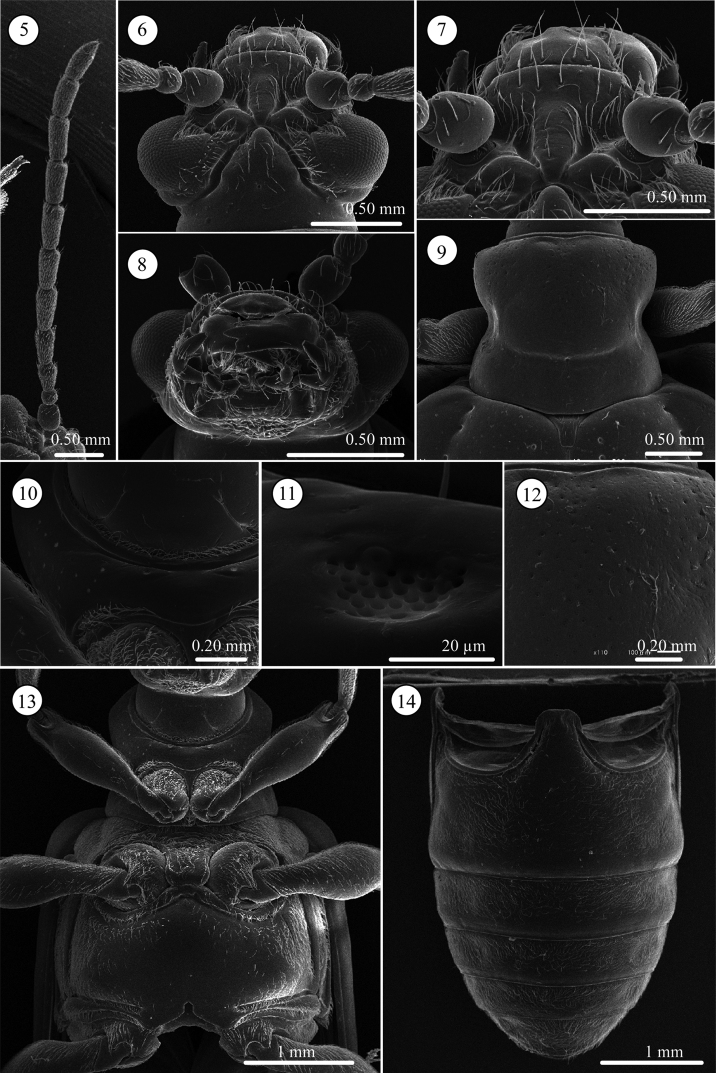
*Lema diversipes*. **5** left antenna **6** head, dorsal view **7** frontoclypeus, dorsal view **8** mouth parts **9** prothorax, dorsal view **10** frontal area of prothorax with lotus-seeds-like structure, ventral view **11** lotus-pod like structure, enlarged **12** prothorax with fine punctures, dorsal view **13** thorax, ventral view **14** abdomen, ventral view.

**Head** (Figs 5–7). Slightly longer than wide; vertex not so raised, coarsely covered with relatively long setae, its surface smooth, with shallow fovea on top, in some cases, fovea longitudinally elongate; area between X-shaped vertex groove and compound eye bearing relatively long setae; orbital area triangular, very densely covered with pubescence; frontal tubercle glabrous; frontoclypeus triangular, bearing relatively long setae, central region glabrous; labrum with ca. Eight relatively long setae, anterior margin corrugated, medial part anteriorly projected; antenna filiform, ca. 0.6 times as long as body length, antennomeres 1 and 2 subglobular and almost glabrous with a few setae, antennomeres 3–11 bearing velutinous setae, antennomeres 5–11 each ringed along apex by a few long setae, antennomere 3 slightly shorter than 4, antennomere 5 slightly longer than 6, antennomeres 6-10 subequal in length, antennomeres 3–10 elongate but slightly thickening apically, apex of antennomere 11 conically prominent.

**Pronotum** (Figs 9 and 12). Subequal in width and length, laterally constricted in middle; surface sparsely, coarsely punctate also micropunctate between larger punctures; transverse groove present near base with fovea medially; anterior and basal margins narrowly margined, basal margin densely pubescent; a long seta present in anterior and posterior angles.

**Scutellum** (Fig. 9). Trapezoidal and relatively longitudinally elongated, posterior angles round, in some specimens posterior margin completely rounded; sparsely pubescent.

**Elytra** (Figs 1–2). 1.7 times longer than wide; very slightly depressed on anterior region in Taiwanese individuals but not depressed in the holotype. Lateral margin parallel; punctures slightly weakening posteriorly, interspaces smooth and slightly raised on apical ⅓.

**Pygidium**. Anterior ⅓ densely covered with short hair-like projections except for stridulatory organ in anterior middle; posterior ⅔ with dense, stout setae.

**Palpi of mouth parts** (Fig. 8). Apical palpomere of maxillary palpi relatively slender, conico-cylindrical; other palpomeres elongate, tapering basally. Labial palpi with four palpomeres, apical three palopmeres relatively stout, apical palpomere conico-cylindrical.

**Prothorax**(lateral and ventral, Figs 10, 11, and 13). Anterior part of prosternum transversely oblong, posterior margin covered with pubescence, anterior region glabrous with very fine lotus-pod like structures, some specimens with transverse wrinkles. Prosternal process very narrow and higher than anterior part, with pubescence on ridgeline, widened posteriorly. Surface of pronotal hypomeron smooth. Posterior arms of pronotal hypomeron not closed and forming arms, but prosternal process bridges them. Anterior and posterior margins of prothorax with pubescent fringe; anterior margin fringed with two rows of setae; anterior margin with curved and straight setae and posterior margin with one straight seta.

**Mesothorax** (Fig. 13). Surface of mesosternum with deep transverse wrinkles, posterior ½ with pubescence; posterior process with small ridge along posterior margin, its surface covered with relatively long setae. Mesepisternum and mesepimeron entirely covered with dense pubescence.

**Metathorax** (Fig. 13). Metasternum oblong; almost all margins with ridge; surface of median ½ glabrous except margin sparsely covered with relatively long setae; lateral ½ densely pubescent; ridgeline of posterior ridge with dense pubescence. Metepisternum with dense pubescence, lateral ⅓ with glabrous area.

**Legs** (Fig. 13). Procoxae conico-cylindrical, with dense and relatively long pubescence, protrochanters glabrous except with relatively long setae on anterior ridgeline; profemora almost glabrous except lateral apex with dense pubescence ventrally, in dorsal view with relatively dense pubescence except for basal ½ of inner margin glabrous. Mesocoxae spherical, with dense, relatively long pubescence on lower anterior ½; mesotrochanters glabrous except with a few long setae on posterior ridgeline; meso- and metafemora with dense pubescence in ventral view, glabrous except for lateral ^1^⁄_5_ with dense pubescence in dorsal view; metatrochanters glabrous except with a few short setae on posterior ridgeline; meso- and metatibiae slender and uniform in shape, covered with dense pubescence, basal ½ with slightly curved pubescence, apical ½ with transparent straight, stiff pubescence, lateral margin of its apex bordered with translucent brown spines, and armed with two subequal black-brown very short spines on ventral margin.

**Abdominal sterna** (Fig. 14). Surface almost entirely densely covered by short pubescence except posterior margin of sterna 1-4 glabrous; lateral area near base of sternite one with more or less glabrous patch, middle of lateral region more or less depressed.

**Figures 15–17.**
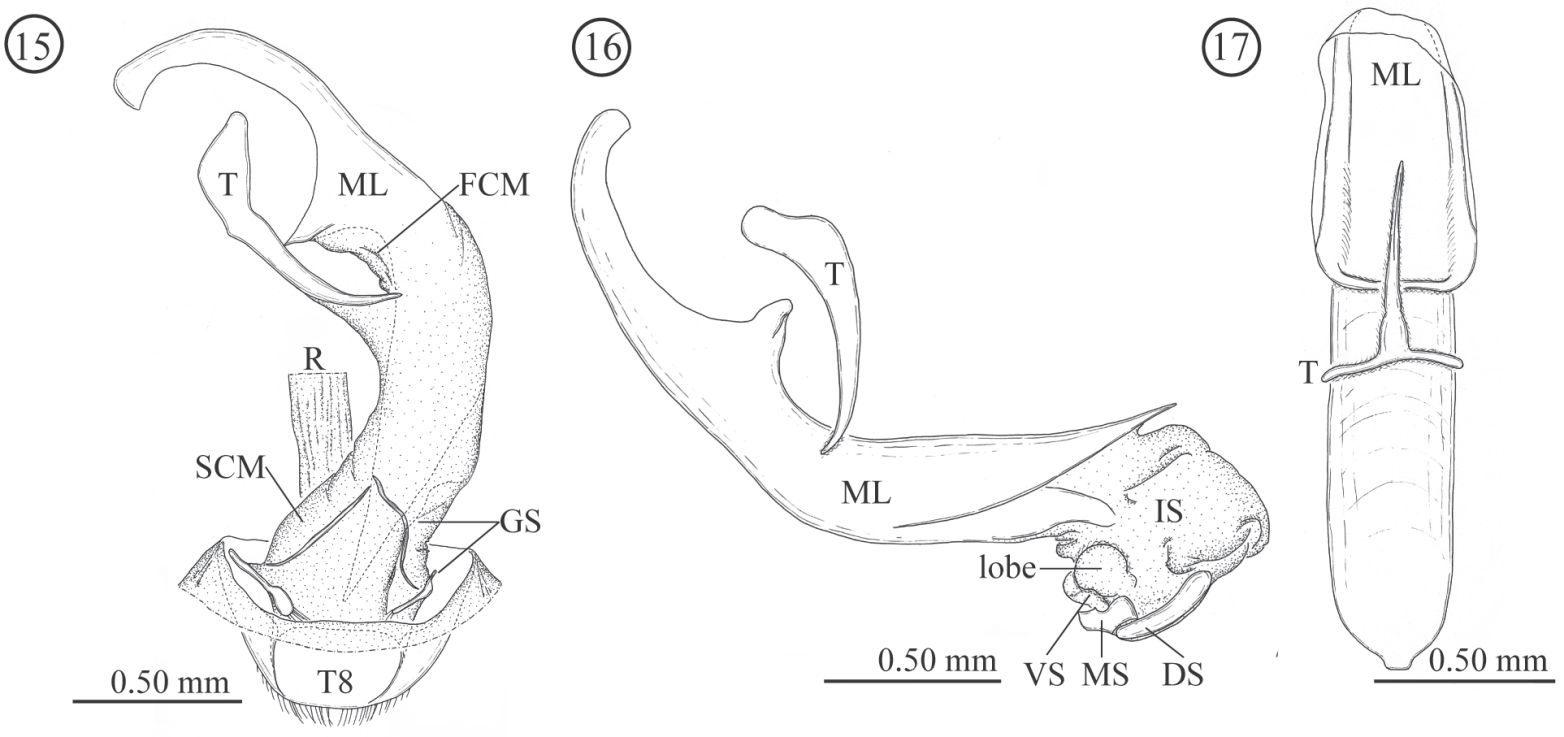
Male genitalia of *Lema diversipes*. **15** entire genitalia in ventral view **16** aedeagus in lateral view, right side corresponds to posterior end **17** median lobe and tegmen in ventral view; DS: dorsal sclerite; FCM: first connecting membrane; GS: gastral spiculum; IS: internal sac; ML: medial lobe; MS: median sclerite; R: rectum; SCM: second connecting membrane; T: tegmen; T8: tergite 8; VS: ventral sclerite.

**Male genitalia** (Figs 15–17). Consisting of five parts: tergite 8, gastral spiculum, tegmen, median lobe and internal sac. Tergite 8 similar to that of the female as described below. Gastral spiculum consisting of two pairs of twig-like sclerites, one pair longer than the other, shorter pair asymmetrical and spoon-like in ventral view. Basal piece of tegmen triangular with rounded corner in lateral view, tapering toward base. Median lobe relatively slender, median foramen expanding and occupying ⅓ of ventral surface in lateral view, ventral end of median orifice with rectangular protrusion which has rounded corner. Internal sac without flagellum and pocket as observed in *Lema lacertosa*; having dorsal-, median-, and ventral sclerites; dorsal, median and ventral sclerites block-like; ventral sclerite covered with a pair of rounded lobes formed by a membrane.

**Figures 18–23.**
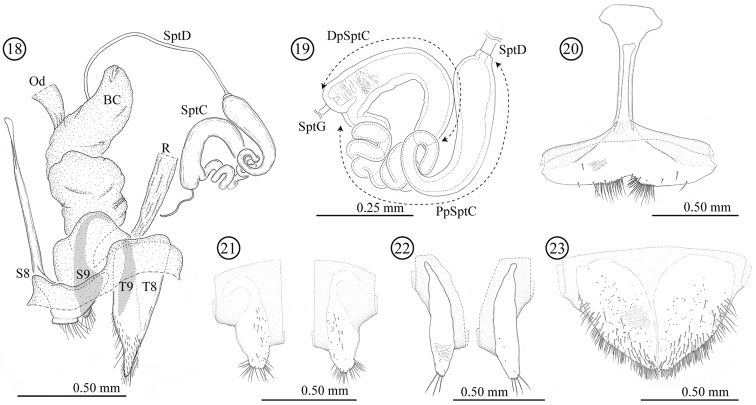
Female genitalia of *Lema diversipes*. **18** entire female genitalia and a part of reproductive system in lateral view **19** spermathecal capsule **20** sternite 8 **21** sternite 9 **22** tergite 9 **23** tergite 8; BC: bursa copulatrix; Od: oviduct; R: rectum; SptD: spermathecal duct; SptG: spermathecal gland; SptC: spermathecal capsule; PpSptC: proximal part of spermathecal capsule; DpSptC: distal part of spermathecal capsule; S8: sternite 8; S9: sternite 9; T8: tergite 8; T9: tergite 9.

**Female genitalia and a part of reproductive systems** (Figs 18–23). Bursa copulatrix balloon-like with its wall thickened but soft. Spermatheca with relatively long duct (0.79 mm, N = 1), opening to ventral side of bursa copulatrix. Wall of spermathecal capsule well sclerotized and thick; distal part of spermathecal capsule hook-shaped, proximal part strongly coiled; inner surface completely covered by very fine winkle-like sculpture. Genitalia consisting of four parts: tergites 8 and 9, and sternites 8 and 9; sclerotization of tergite 8 gradually weakened toward midline; sternite 8 with stick-like apodeme; tergite and sternite 9 consisting of a pair of sclerites; tergites 8 and 9 largely covered by scale- to winkle-like sculpture, marginal region of tergite 8 covered by relatively stout setae; both sides of the sternite 8 covered by scale-like sculpture; posterior area of sternite 9 weakly wrinkled.

**Measurements**.Elytral length: male: 4.53 ± 0.22 mm (mean ± SD, N = 5), female: 5.14 ± 0.03 mm (N = 2). Elytral width: male: 2.68 ± 0.06 mm, female: 2.92 ± 0.06 mm. Pronotum length: male: 1.44 ± 0.04 mm, female: 1.46 ± 0.07 mm. Pronotum width: male: 1.43 ± 0.05 mm, female: 1.58 ± 0.08 mm.

#### Host plant.

(Figs 43–44) Fabaceae: *Pueraria lobata* (Willd.) subsp.* thomsonii* (Benth.) Ohashi .

#### Distribution.

China, Taiwan (**new record**).

#### Remarks.

**Condition of holotype**. Right side of the head and abdomen in dorsal view with wormholes. Almost all pubescence of the body surface is lost. However punctures remain, which enable us to know setal arrangement. Comparing the arrangement of the punctures and setae in newly collected Taiwanese specimens, we identified the specimens collected in Taiwan as *Lema diversipes* and described the characteristic of setae based on the Taiwanese specimens.

**Justification of resurrection of *Lema diversipes* and removing it from synonymy of from *Lema lacertosa***. *Lema diversipes* was synonymized under *Lema lacertosa* by Gressitt and Kimoto (1961) without explanation, and researchers have followed this treatment (Kimoto and Gressitt 1979, Schmitt 2010, Warchałowski 2011). The two species which we identified as *Lema lacertosa* and *Lema diversipes* in this study clearly differ in their external appearance, the genital structure, and their host plants. Feeding on Fabaceae plants for *Lema diversipes* is very rare in members of the genus *Lema* (Schmitt 1988).

The original descriptions (Lacordaire 1845, Pic 1921) and redescriptions (Baly 1865) of the species are not detailed enough to distinguish them, but the measurements in the original- or re-descriptions differs greatly between them (*Lema lacertosa*: 2 2/3 lin. = 5.64 mm described by Baly 1865, *Lema diversipes*: 8 mill = 8 mm described by Pic 1921). Although we asked curators in the Natural History Museum (London), the Muséum National d’Histoire Naturelle (Paris), the Brussels Museum, the Bishop Museum (Hawaii) and the Museum of Comparative Zoology (Cambridge), we could not find the type specimen of *Lema lacertosa*. However, reading taxonomic papers, we judge that chrysomelid taxonomists have a consensus of the characters distinguishing *Lema lacertosa* (e.g. Gressitt and Kimoto 1961, Kimoto and Gressitt 1979, Warchałowski 2011). In fact, we found Indian *Lema* specimens which were identified as *Lema lacertosa* was quite similar to the smaller Taiwanese *Lema* specimens. We compared generally accepted species as *Lema lacertosa* and the holotype of *Lema diversipes*. The holotype of *Lema diversipes* is clearly distinguished from *Lema lacertosa*, and we judged *Lema diversipes* should be treated as a separate species.

### Lema (Lema) lacertosa

Lacordaire, 1845

http://species-id.net/wiki/Lema_lacertosa

Lema lacertosa Lacordaire, 1845: 339 [Bengal] [type depository unknown]; Baly 1865: 11; Jacoby 1908: 35; Monrós 1959: 186; Gressitt and Kimoto 1961: 69; Kimoto and Gressitt 1979: 249; Schmitt 2010: 362; Warchałowski 2011: 69.
Lema phungi Pic, 1924: 13 [Tonkin, Vietnam] (MNHN); Monrós 1959: 188; Warchałowski 2011: 69 (as probable synonym of *Lema lacertosa*). **synonymy confirmed**
Lema jeanvoinei Pic, 1932: 11 [Hanoi, Vietnam] (MNHN); Monrós 1959: 186; Warchałowski 2011: 69 (as probable synonym of *Lema lacertosa*). **synonymy confirmed**

#### Type series.

*Lema phungi*: lectotype 1 ♀, here designated, labeled: Hoa Binh/ Tonkin// Phungi/ Pic// Muséum Paris/ 1958/ Coll. Pic// SYNTYPE// MNHN/ EC3057//; paralectotype: 1 ♀// Hoa Binh/ / Tonkin// Phungi/ Pic// Museum Paris/ 1958/ Coll. Pic// SYNTYPE// MNHN/ EC3058//.

*Lema jeanvoinei*. 1 ♀// Tonkin/ Hanoi/ 7. IV. 1918/ JEANVOINE// Dessous/ et/ membres/ largement/ noirs// jeanvoinei/ n sp// Museum Paris/ 1958/ Coll. Pic// HOLOTYPE// MNHN/ EC3059//.

#### Material examined.

**Taiwan**: 4 exs.: Chiayi, Minhsiung, 29.IV.2010, leg. W.-T. Liu (TARI); 2 exs.: Chiayi, Niutoulun, 30.III.2010, leg. W.-T. Liu (TARI); 2 exs.: Kaoshiung, Niaosung, 2.VI.2008, leg. C.-H. Liu (TARI); 1 ex.: Kaoshiung, Taiao, 8.IX.2008, leg. W.-T. Liu (TARI); 1 ex.: Nantou, Chichi, 19.VIII.2010, leg. W.-T. Liu (TARI); 5 exs.: Pingtung: Chaochou, 5.XI.2009, leg. J.-C. Chen (TARI); 3 exs., same locality, 16.III.2010, leg. M.-H. Tsou (TARI); 11 exs., same locality, 2.VI.2010, leg. M.-H. Tsou (2 exs. in SEHU; 9 exs. in TARI); 3 exs.: Pingtung, Hengchun, 9.VII.2011, leg. J.-C. Chen (TARI); 1 ex.: Pingtung, Kaoshih, 8.V.2012, leg. J.-C. Chen (TARI); 1 ex.: Pingtung, Nanjen Lake, 3.I.2011, leg. J.-C. Chen (TARI); 1 ex., same locality, 29.IV.2011, leg. J.-C. Chen (TARI); 5 exs.: Pingtung, Wukoushui, 21.VIII.2010, leg. J.-C. Chen (TARI); 1 ex.: Taipei, Kuantu, 15.IX.2010, leg. S.-F. Yu (SEHU); 2 exs., same locality, 8.VI.2011, leg. S.-F. Yu (TARI); 1 ex.: Taitung, Anshuo, 7.XI.2011, leg. J.-C. Chen (TARI); 3 exs.: Taoyuan, Meihuali, 14.VII.2010, leg. H. Lee (SEHU); 5 exs.: Taoyuan, Chungli, 19.X.2010, leg. H. Lee (TARI); 4 exs.: Taoyuan, Kuanyin, 16.VI.2010, leg. M.-H. Tsou (TARI). **Malaysia:** 3 exs.: Negeri Selangor, Ulu Gomback, (Univ. Malaysia Field Studies centre. 220 m alt.), 10.III.2009, leg. Y. Matsumura (SEHU); 1ex.: Negeri Selangor, Ulu Gomback (Univ. Malaysia Field Studies/ centre. 220 m alt.) 8.XI.2009, leg. Y. Matsumura (SEHU); 1ex: Jalan Pahang Perk, Batu 19 (570m alt.), 8.XI.2009, leg. Y. Matsumura et al. (SEHU). **India:** 2 exs.: Calcutta, 14-19.X.1978, leg. JAP-IND CO TR (SEHU).

#### Diagnosis.

*Lema lacertosa* can be separated from *Lema diversipes* by the following combination of characters: body is distinctly smaller; anterior margin of the clypeus is curved inward and slightly concave; posterior lines of the vertex grove nearly straight; anterior region of the ventral surface is nearly black and posterior ⅓ (sterna 2-5) orange to brown; sterna almost entirely covered by pubescence, except around midline of the sternum 1 glabrous.

#### Redescription.

**Body coloration** (Figs 3–4). Dorsum: Labrum and anterior ½ of frontoclypeus black, antenna brownish-black except antennomeres 1 and 2 which are orange to brown; remaining part of head, pronotum, scutellum and elytra brownish to reddish-orange. Procoxae black, protrochanters brown, profemora, protibiae, and protarsi orange with diffuse brown to blackish line; meso- and metatrochanters brown, femora, tibiae, and tarsi of meso- and metalegs black. Venter: anterior ⅓-½ of prothorax orange, remaining area black to brownish-black; meso- and metathorax black; first abdominal sternite black to blackish-brown, other sterna orange to brown. Pubescence white. Antenna lighter colored than other parts, protrochanter and apical section of procoxae orange; proleg black basally. Basal ½ of first abdominal sternite black; especially in Malaysian populations with brighter orange color.

**Figures 24–31.**
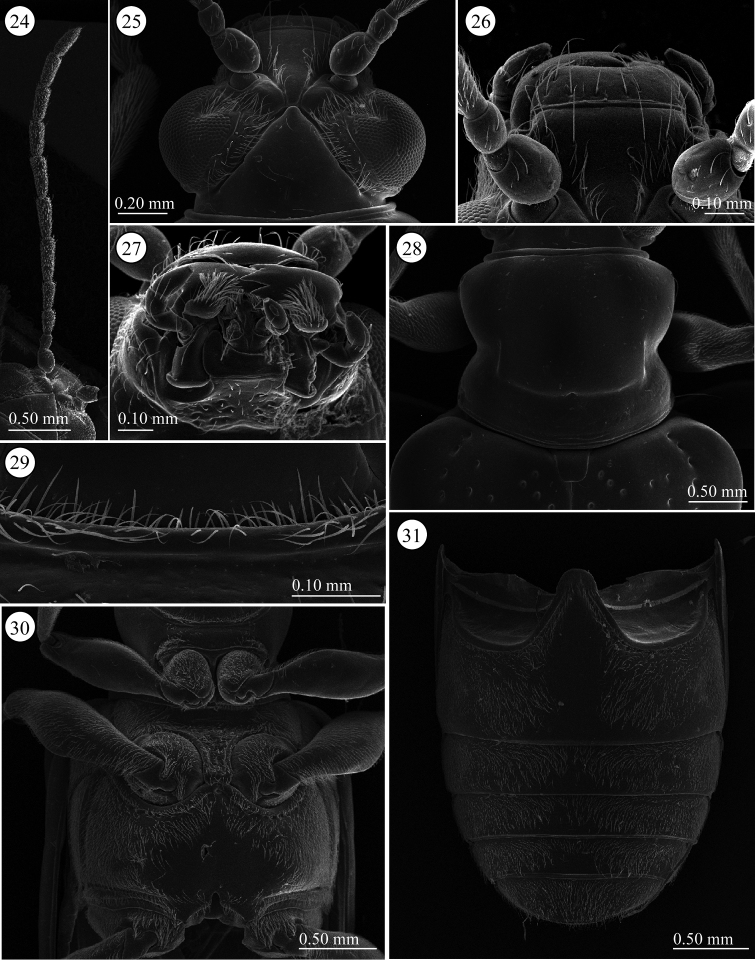
*Lema lacertosa*. **24** left antenna **25** head, dorsal view **26** frontoclypeus, dorsal view **27** mouth parts **28** prothorax, dorsal view **29** frontal fringe of prothorax, ventral view **30** thorax, ventral view **31** abdomen, ventral view.

**Head** (Figs 24–26). Width and length almost equal; vertex not raised, glabrous, surface smooth; area between X-shaped vertex groove and compound eye with relatively long setae, covered with fine sculpture; orbital area triangular, densely covered with pubescence; frontal tubercle indistinct, glabrous; frontoclypeus triangular, covered with setae, setae relatively dense on posterior ½, medial line region glabrous; labrum with ca. Seven relatively long setae, anterior margin curved inward and slightly concave; antenna filiform, ca. 0.7 times as long as body length, antennomeres 1–2 subglobular and almost glabrous with a few setae, antennomeres 3-11 bearing velutinous pubescence, apex of antennomeres 5–11 ringed with a few long setae, antennomere 3 subequal in length to 4, antennomeres 3+4 slightly longer than 5, antennomere 4 or 5 longest depending on individuals, antennomeres 6-10 subequal in length, antennomeres 3-10 cylindrical slightly thickening apically, apex of antennomere 11 conically prominent.

**Pronotum** (Fig. 28). Slightly wider than long to almost equal, laterally constricted at middle; surface with a few small punctures around midline and anterior angles, rest with very fine punctures, transverse groove present near base with fovea in middle, anterior and posterior margins narrowly margined, posterior ridge internally with dense short setae. A long seta present in each anterior and posterior angle.

**Scutellum** (Fig. 28). Trapezoidal and relatively wide, posterior margin concave, indistinct in some specimens. Surface glabrous, but in three of five Taiwanese specimens covered with a few setae.

**Elytra** (Figs 3–4). 1.7 times longer than wide; one of six Taiwanese specimens very slightly depressed anteriorly but not depressed or indistinctly impressed in the other specimens. Lateral margins parallel; punctures slightly weakening posteriorly.

**Pygidium**. Anterior ⅓ densely covered with short hair-like projections except for stridulatory organ in anterior middle, size of stridulatory organ relatively small; posterior ⅔ with dense, stout setae.

**Palpi of mouth parts** (Fig. 27). Apical maxillary palpomere relatively stout and conico-cylindrical but not enlarged; other palpomeres cylindrical, narrowing basally; one of two Indian specimens examined with relatively slender apical palpomere. Labial palpi with four palpomeres, apical three palpomeres relatively stout but not enlarged, apical palpomere conico-cylindrical.

**Prothorax**(lateral and ventral, Figs 29–30). Anterior area of prosternum transversely oblong anteriorly, with pubescent patch posteriorly, glabrous anteriorly, some specimens with very weak transverse wrinkles. Prosternal process very narrow and not raised, widened posteriorly. Surface of pronotal hypomeron smooth. Posterior arms of pronotal hypomeron normally not closed in most specimens, but closed in one Malaysian specimen and fused in one Indian specimen; prosternal process with bridge arms, bridge relatively short and not completely covering arms. With pubescent fringe anteriorly and posteriorly; anterior margin fringed with two rows of setae.

**Mesothorax** (Fig. 30). Surface of mesosternum with fine sculpture and pubescence; posterior process with ridge along margin, pubescence on posterior ridge relatively long. Mesepisternum and mesepimeron with dense pubescence.

**Metathorax** (Fig. 30). Metasternum oblong; almost entire margin with ridge; surface of medial area glabrous and other areas covered with pubescence; medial part of anterior ridge with relatively long pubescence; posterior margin between metacoxae with curved pubescence. Metepisternum with dense pubescence, lateral ⅓ with glabrous area overlapping elytra.

**Legs**. Procoxae conico-cylindrical, densely covered with pubescence, protrochanters glabrous, with relatively long setae on anterior ridgeline; profemora nearly glabrous except apex laterally with pubescence ventrally, dorsum with relatively dense pubescence except for glabrous base. Mesocoxae spherical, densely pubescent on lower anterior ½; mesotrochanters glabrous with very long pubescence on posterior ridgeline; meso- and metafemora with dense pubescence ventrally, glabrous dorsally except for dense pubescence apically. Metacoxae pubescent; metatrochanters glabrous except with long pubescence on posterior ridgeline; tibiae slender and only slightly tapering apically, covered with dense pubescence, basally ⅓ to ½ with slightly curved pubescence, apically with straight, transparent setae, almost glabrous dorsally; tibiae with lateral margin bordered with translucent brown spines apically, and armed with pair of very short, subequal, black-brown spines ventrally.

**Abdominal sterna** (Fig. 31). Surface almost entirely densely covered by short pubescence; only around midline of sternite one glabrous, some specimens more or less depressed laterally.

**Figures 32–36.**
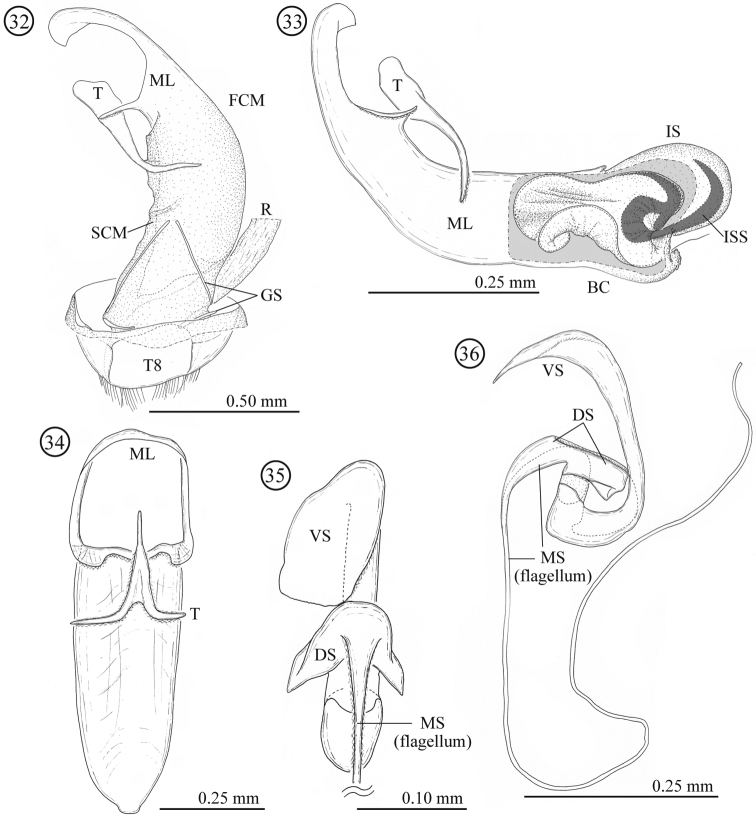
Male genitalia of *Lema lacertosa*. **32** entire genitalia in ventral view **33** aedeagus in lateral view; outer membrane of internal sac was partly removed. Right side corresponds to posterior end **34** median lobe and tegmen in ventral view **35** internal-sac sclerites in dorsal view, basal part is enlarged **36** internal-sac sclerites in lateral view; Bc: body cavity; DS: dorsal sclerite; FCM: first connecting membrane; GS: gastral spiculum; IS: internal sac; ISS: internal-sac sclerites; ML: median lobe; MS: median sclerite; R: rectum; SCM: second connecting membrane; T: tegmen; T8: tergite 8; VS: ventral sclerite.

**Male genitalia** (Figs 32–36). Consisting of five parts: tergite 8, gastral spiculum, tegmen, median lobe and internal sac. Tergite 8 similar to that of female as described below. Gastral spiculum consisting of two pairs of twig-like sclerites, one pair longer than the other. Basal piece of tegmen rectangular in lateral view, tapering toward base. Median lobe stout, median foramen expanding and occupying ⅓ of ventral surface in lateral view, ventral end of median orifice round with rectangular and rounded protrusion. Internal sac with specialized state as in many members of the subgenus *Lema*, i.e. having pocket for storing elongated flagellum; median and ventral sclerites forming flagellum (1.58 mm, N=1); dorsal sclerite not separated.

**Figures 37–42.**
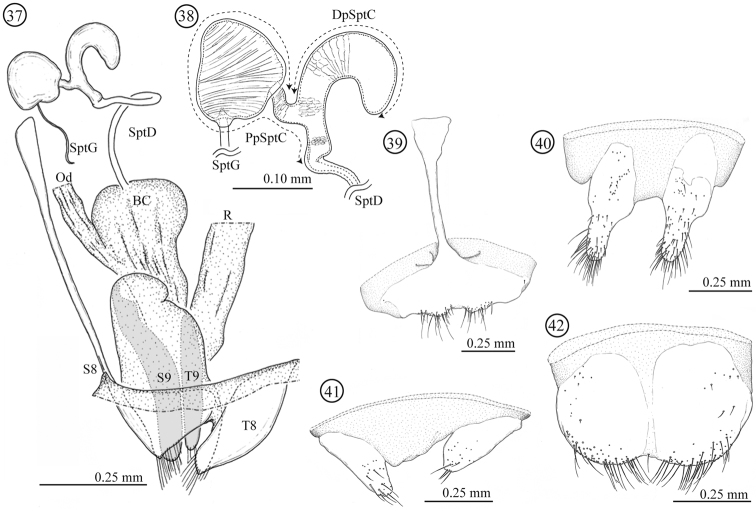
Female genitalia of *Lema lacertosa*. **37** entire female genitalia and a part of reproductive system in lateral view **38** spermathecal capsule **39** sternite 8 **40** sternite 9 **41** tergite 9 **42** tergite 8; BC: bursa copulatrix; Od: oviduct; R: rectum; SptD: spermathecal duct SptG: spermathecal gland; SptC: spermathecal capsule; PpSptC: proximal part of spermathecal capsule; DpSptC: distal part of spermathecal capsule; S8: sternite 8; S9: sternite 9; T8: tergite 8; T9: tergite 9.

**Female genitalia and a part of reproductive systems** (Figs 37–42). Spermathecal duct relatively long (0.36–0.49mm, N = 2) with no specialized structure in opening to bursa copulatrix. Spermathecal capsule well sclerotized, its wall relatively thick; distal part hook-shaped, inner surface covered by winkle-like sculpture, junction area to spermathecal duct covered by scale-like sculpture; proximal part with a large potato-like structure, inner surface covered by transverse winkles. Spermathecal gland opening on a light-bulb like structure. Genitalia of four parts: tergites 8 and 9, and sternites 8 and 9; tergites 9 and sternite 9 consisting of a pair of sclerites; sclerotization of tergite 8 gradually weakened toward midline; sternite 8 with stick-like apodeme; posterior area of sternite 8 covered by scale-like sculpture; upper area of tergite 8 weakly covered by scale-like sculpture and lower area with fine pointed projections.

#### Measurements.

**Specimens collected from India.** Elytral length: male: 3.04 mm (N=1), female: 3.38 mm (N=1). Elytral width: male: 1.77 mm, female: 2.00 mm. Pronotum length: male: 1.00 mm, female: 1.15 mm. Pronotum width: male: 1.04 mm, female: 1.27 mm.

**Specimens collected from Taiwan.** Elytral length: male: 3.36 ± 0. 21 mm (mean ± SD, N=2), female: 3.56 ± 0.15 mm (N=4). Elytral width: male: 1.96 ± 0.11 mm, female: 2.05 ± 0.13 mm. Pronotum length: male: 1.08 ± 0.05 mm, female: 1.09 ± 0.05 mm. Pronotum width: male: 1.15 ± 0.02 mm, female: 1.20 ± 0.07 mm.

**Specimens collected from Malaysia.** Elytral length: male: 3.15 mm (N=1), female: 3.57 ± 0.21 mm (N=4). Elytral width: male: 1.81 mm, female: 2.13 ± 0.14 mm. Pronotum length: male: 0.96 mm, female: 1.10 ± 0.03 mm. Pronotum width: male: 1.13 mm, female: 1.21 ± 0.05 mm.

#### Host plant.

(Figs 45–46) Commelinaceae: *Commelina communis* L., 1753.

**Figures 43–48.**
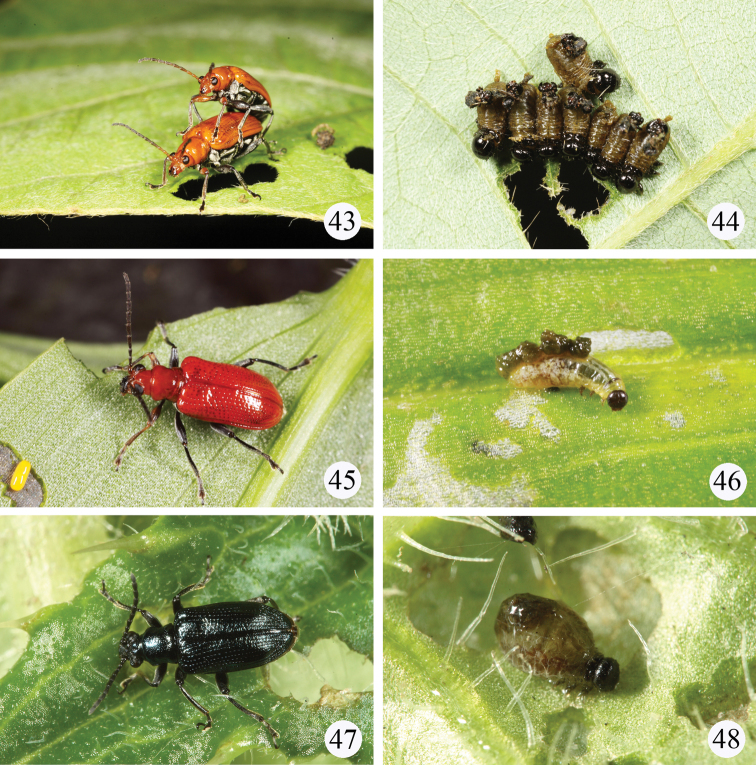
Live adults or larvae in the field. **43–44** *Lema diversipes* on *Pueraria lobata* (Willd.) subsp.* thomsonii* (Benth.) Ohashi **43** a pair of adults **44** a cluster of larvae **45–46** *Lema lacertosa* on *Commelina communis* L. **45** adult **46** larva **47–48** *Lema cyanella* on *Cirsium japonicum* **47** adult **48** larva.

#### Distribution.

India, Malaysia, and Taiwan (**new record**). This species is also recorded from Laos, Vietnam, S. China, and Singapore (Kimoto and Gressitt 1979), and Nepal (Schmitt 2010). These identifications require confirmation.

#### Remarks.

**Justification of identification of *Lema lacertosa*.** Although Kimoto and Gressitt (1979) stated the type depository, they did not observe types and the type could not be located (see also remarks under *Lema diversipes*). However from investigation of the literature we judged that there is a consensus for the identity of *Lema lacertosa* among chrysomelid taxonomists. Features of the commonly accepted species have no contradiction with the original description and the specimens which we examined and identified as *Lema lacertosa*.

Although we could not locate the holotype of *Lema lacertosa*, we have no evidence regarding the disappearance of the holotype. In addition, the identity of this species is relatively stable, so we do not designate a neotype for this species.

### Lema (Lema) cyanella

(Linnaeus, 1758)

http://species-id.net/wiki/Lema_cyanella

#### Material examined.

Taiwan: 4 exs.: Taipei, Juifang, 10.II.2010, leg. H. Lee (TARI); 1ex., same locality, 21.II.2010, leg. M.-H. Tsou (TARI); 2 exs., same locality, 6.III.2010, leg. M.-H. Tsou (TARI); 11 exs., same locality (= Nanya trail), 15.III.2010, leg. H. Lee (TARI); 2 exs., same locality, 19.III.2010, leg. H. Lee (TARI); 5 exs., same locality, 20.III.2010,leg. H. Lee (TARI); 8 exs., same locality, 1.IV.2010, leg. M.-H. Tsou (TARI); 1 ex., same localilty, 18.IV.2010, leg. M.-H. Tsou (TARI); 1 ex., same locality, 25.IV.2010, leg. M.-H. Tsou (TARI); 2 exs.: Taipei, Pinglin, 1.VII.2008, leg. H. Lee (TARI).

#### Remarks.

This species was redescribed by Matsumura et al. (2011) who presumed *Cirsium japonicum* DC and *Lema suffultum* Matsum. (Asteraceae) to be its host plants. Here *Cirsium japonicum* (Figs 47–48) is confirmed as a host plant by field observations and laboratory rearing.

#### Distribution.

Europe, China, Mongolia, Korea, Taiwan (**new record**), Japan.

### Lema (Petauristes) solani

Fabricius, 1798

http://species-id.net/wiki/Lema_solani

#### Material examined.

**Taiwan**: 19 exs.: Chiayi, Chungpu, VIII.2007, leg. H.-T. Shih (TARI); 1 ex.: Hsinchu, Mamei, 4.V.2008, leg. S.-F. Yu (TARI); 1 ex.: Nantou, Wanfengtsun, 21.IV.2007, leg. W.-T. Liu (TARI); 1 ex.: Taichung, Pahsienshan, 5.VIII.2007, leg. W.-T. Liu (TARI); 4 exs.: Taichung, Tahsuehshan, 1.V.2012, leg. W.-T. Liu (TARI); 3 exs.: Tainan, Danei, 9.VII.2007, leg. W.-T. Liu (TARI); 1 ex.: Tainan, Meiling, 12.III.2011, leg. M.-L. Jeng (TARI); 2 exs.: Taipei, Sanchih, 7.VIII.2011, leg. C.-C. Cheng (TARI); 5 exs., same locality, 13.VIII.2011, leg. H. Lee (TARI); 2 exs.: Taoyuan, Luchu, 16.VI.2009, leg. W.-T. Liu (TARI).

#### Remarks.

**Figures 49–52.**
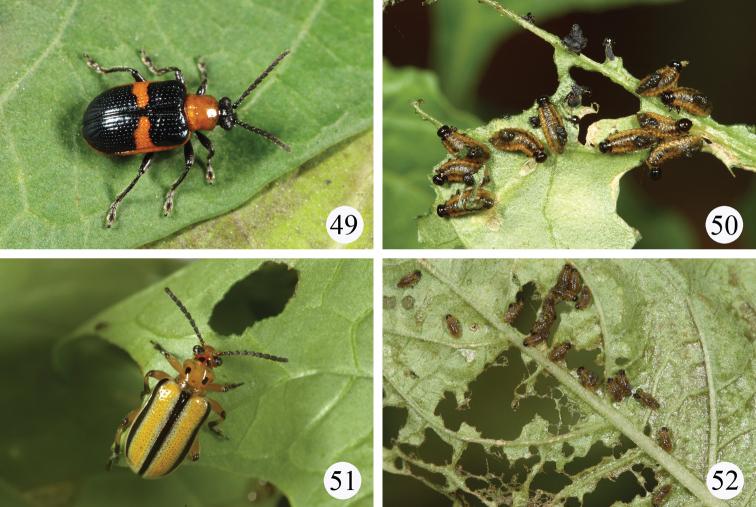
Live adults and larvae in the field. **49–50** *Lema solani* on *Solanum americanum* **49** adult **50** larvae **51–52** *Lema trivittata trivittata* on *Physalis angulata* **51** adult **52** larvae.

Lee and Cheng (2007) were the first to record this species from Taiwan although it is an introduced species originally distributed from the Eastern United States to Texas (White 1993). Species of the genus *Solanum* (Solanaceae) were reported as its host plants in the US. Adults and larvae were found feed on leaves of *Solanum americanum* Miller (Figs 49–50) in Taiwan. *Solanum americanum* is also an introduced species for Taiwan.

#### Distribution.

Eastern United States to Texas, Taiwan.

### Lema (Petauristes) trivittata trivittata

Say, 1824

http://species-id.net/wiki/Lema_trivittata_trivittata

#### Material examined.

**Taiwan**: 2 exs.: Taitung, Lanyu island, 5.V.2012, leg. S.-F. Yu (TARI); 16 exs.: Yunlin, Pichiao, 16.VI.2010, leg. H. Lee (TARI); 2 exs., same locality (= Tuku), 30.VI.2010, leg. H. Lee (TARI); 2 exs., same locality, 9.VII.2010, leg. H. Lee (TARI).

#### Remarks.

This species is also an introduced one for Taiwan because its original distribution is limited to the United States and Canada (White 1993). Recently Aoyagi (2012) and Kawaji (2012) reported occurrence of *Lema trivittata* from Miyako and Iriomote islands of Japan. Although they did not mention the subspecies name, from the pictures in the papers we considered that they are same subspecies. Considering the geographic placement of the two islands and Taiwan, the origin of invasion could be once and rapidly spread.

Beetles were found to feed on leaves of *Physalis angulata* L. (Figs 51–52) and *Physalis peruviana* L. in Taiwan. Both plants are introduced species for Taiwan originally from the United States. Aoyagi (2012) also mentioned that the probable host plant is *Physalis angulata* L. in Miyako island of Japan and warned that it is a potential pest for cultivation of a leaf tabacco which is one of popular cultivation in the island.

#### Distribution.

United States and southern Canada, Taiwan (**new record**), and Japan (Ryukyu: Miyako and Iriomote islands).

##### Key to species of the genus *Lema* from Taiwan (modified after Kimoto and Takizawa 1997)

A key to 11 known species of *Lema* from Taiwan was presented by Kimoto and Takizawa (1997). Of these species, *Lema postrema* Bates, 1866 is a junior synonym of *Lema fortunei* Baly, 1859 (Schmitt 2010); *Lema coromandeliana* (Fabricius, 1798) is a junior synonym of *Lema praeusta* (Fabricius, 1792) (Warchałowski 2011).

**Table:** 

1	Elytron without scutellar row of punctures	(subgenus *Petauristes*) 2
–	Elytron with a short scutellar row of punctures	(subgenus *Lema*) 7
2	Elytron blackish-blue	3
–	Elytron yellowish-brown	5
3	Entire elytron blackish-blue	4
–	Lateral margin of elytra yellow, with transverse yellow band at middle	*Lema solani* Fabricius
4	Generally reddish-brown except antenna black	*Lema fortunei* Bates
–	Generally black except head and prothorax reddish-brown	*Lema honorata* Baly
5	Entire elytra yellowish	*Lema pectoralis* Baly
–	Elytra with black spots or stripes	6
6	Elytra with three black, longitudinal stripes	*Lema trivittata trivittata* Say
–	Elytra with large, black spots at base and subapex; in some specimens entire elytra black except apex	*Lema koshunensis* Chûjô
7	Mesotibia with a distinct denticulation in middle	*Lema coronata* Baly
–	Mesotibia without distinct denticulation	8
8	Pronotum with two transverse furrows at basal ½	*Lema praeusta* (Fabricius)
–	Pronotum with one transverse furrow at basal ½	9
9	Generally blackish-blue	10
–	Generally yellowish-brown	12
10	Pronotum without punctures	*Lema cyanea* Fabricius
–	Pronotum with punctures	11
11	Abdominal ventrites III-V yellowish-brown, vertex relatively flat	*Lema concinnipennis* Baly
–	Abdominal ventrites blackish-blue, vertex swollen	*Lema cyanella* (Linneaus)
12	Middle and hind legs black except front femur reddish-brown	13
–	All legs yellowish-brown	14
13	Body longer (7.5–8.2 mm), abdominal ventrites I-V black (Fig. 2)	*Lema diversipes* Pic
–	Body shorter (4.5–5.5mm), abdominal ventrites II-V reddish-brown (Fig. 4)	*Lema lacertosa* Lacordaire
14	Elytron with basal and postmedian black spots	*Lema esakii* Chûjô
–	Entire elytron yellowish-brown	15
15	Elytron with transverse furrow behind humerus; vertex without pubescence	*Lema rufotestacea* Clark
–	Elytron without transverse furrow; vertex with dense pubescence	*Lema coomani* Pic

## Supplementary Material

XML Treatment for Lema (Lema) diversipes

XML Treatment for Lema (Lema) lacertosa

XML Treatment for Lema (Lema) cyanella

XML Treatment for Lema (Petauristes) solani

XML Treatment for Lema (Petauristes) trivittata trivittata
